# A Systematic Literature Review of Strategies Implemented in Extended Education Settings to Address Children’s Mental Health and Wellbeing

**DOI:** 10.1007/s10567-024-00494-3

**Published:** 2024-07-14

**Authors:** Sarah Murray, Sonja March, Yosheen Pillay, Emma-Leigh Senyard

**Affiliations:** 1 Centre for Health Research, University of Southern Queensland, Springfield, Australia; 2 School of Psychology and Wellbeing, University of Southern Queensland, Springfield, Australia; 3 School of Education, University of Southern Queensland, Springfield, Australia; 4 Manna Institute, University of Southern Queensland, Springfield, Australia

**Keywords:** Outside school hours care, Educators, Wellbeing, Mental health, Children, Extended education

## Abstract

Mental health and wellbeing problems in middle childhood are increasing worldwide which needs more support than just clinical services. Early intervention has been explored in other settings, but not in extended education care settings such as outside school hours care (OSHC). A systematic literature review was undertaken to determine what interventions have been tested in extended education settings to address or promote emotional, behavioural, or social wellbeing in children, and to assess how effective they have been. A PRISMA guided search found seven peer reviewed articles from an initial pool of 458. Data from the articles were extracted and the mixed method appraisal tool (MMAT) was applied to assess methodological quality of the studies design, data collection, and analyses. The final selections were methodologically heterogeneous with an average MMAT quality rating of 71%. All but one of the interventions were delivered to children in small group settings and were a mix of activities. Studies that trained educators to deliver the interventions were limited and no data were collected for them. The two interventions that trained educators to deliver content to children were seen as promising. This review showed an overall paucity of research examining interventions delivered in extended education settings to improve children’s wellbeing. Given variations in extended education services and the absence of formal qualifications required for educators, further research is needed to understand what interventions may be effective and what role educators could play in such interventions or in supporting children’s wellbeing in extended education.

This review protocol was prospectively registered with PROSPERO. Registration ID: CRD42023485541 on 03/12/2023.

## Introduction

Outside school hours care (OSHC) services are the fastest growing childcare services in Australia (Cartmel, 2019; Social Research Centre, 2022), providing care to primary school-aged children before and after school, and during school holidays. OSHC is also known by other names internationally, such as school-aged care (SAC) (Department of Education Employment and Workplace Relations, 2011), School-Aged Educare (Klerfelt & Haglund, 2014), Extended Education (Bae, 2019), or outside school time (OST) (Malone, 2017). In some countries, including Australia these services are typically run by organisations external to the school, but occur on school grounds. Considering the wide naming variations, the international research community has broadly adopted the term ‘extended education’ to define the interdisciplinary field of research (Bae, 2019) and this term will be used hereafter. This term refers to settings that (a) include intentionally organised activities, learning, and/or developmental programs, (b) incorporate teaching, learning, and/or development that occurs between adult professionals and young people, (c) occurs outside of school time, such as before and after school, and on school holidays, (d) mostly occurs in a school setting, and is voluntary to attend (Bae & Kanefuji, 2018).

For example, USA-based extended education services are typically seen as places for enrichment with specialised offerings for children (e.g., technology, academic, or art clubs) (Minney et al., 2019) or free play opportunities that allow social-emotional skill development (Noam & Triggs, 2018). In Nordic and Central European countries such as Sweden, Iceland and Germany, after school care is integrated into formal education so that children attend all day schools that cater for learning in formal and informal settings (e.g. Fischer et al., 2014). These settings, researchers believe, contribute to children’s learning and wellbeing as well as to their formal education and potentially, to society as a whole (Klerfelt & Stecher, 2018).

Variations also exist in international social, political, historical and educational needs of extended education that determine how it is offered in each country (Bae, 2019). Researchers in this field highlight the importance of learning that does not occur in the formal educational space of school (Stecher, 2019) and where childhood development and social-emotional learning is valued (Bae, 2019; White et al., 2022). A growing body of evidence shows the social-emotional benefits to children who attend extended education (Durlak & Weissberg, 2007) and that building these skills contribute to long-term academic and life success (Noam & Triggs, 2018). It is therefore important that educators in extended education settings are equipped to support children’s mental health and wellbeing development.

Worldwide, millions of children attend extended education services, often spending more time with extended education educators than with a classroom teacher. In Australia, nearly half a million children (25% of the 5–11 year old population) attend extended education (Social Research Centre, 2022) while in the US 35% of all children aged 6–13 years attend these services (Administration for Children and Families (DHHS) Office of Child Care, 2022a, 2022b). Attendance rates in Europe are similar, with 35% of 6–11 year olds attending extended education services in 29 EU countries and up to 65% of children in some Nordic countries such as Denmark, Slovenia, and Sweden (OECD Family Database, 2022). Despite this, there has been little national or international research interest in the time children spend in extended education.

In Australia, extended education services (OSHC) promote play and leisure for the children in their care, and the pedagogical framework that guides OSHC—My Time, Our Place Framework for School-Age Care in Australia 2.0 (MTOP)—emphasises the importance of children’s development of agency, wellbeing, and social and emotional skills (Australian Government Department of Education (ADGE), 2023). This emphasis on wellbeing is seen internationally as well. For example, the US’ adoption of the Whole School, Whole Community, Whole Child (WSCC) model recognises the social and emotional climate in extended education as an important factor for children to grow up safe, healthy, engaged, challenged, and supported (ASCD & Centers for Disease Control and Prevention, 2014). Some European countries have also enshrined social, emotional, and wellbeing development in policy, such as Scotland’s national approach—Getting it Right for Every Child—which underpins and supports all adults who work with children (including in extended education) to be able to recognise and respond to children’s wellbeing (The Scottish Government, 2019).

Despite the focus on wellbeing in frameworks that govern the implementation of extended education services internationally, little is known in the academic literature about how these educators support the development of children’s social and emotional skills in their day-to-day work, and what interventions have been implemented in extended education services to promote wellbeing or positive mental health. Further, researchers in the emerging field of extended education encourage interdisciplinary studies to better understand children’s outcomes in these settings (Bae, 2019; Stecher, 2019).

### Mental Health in Middle Childhood

The World Health Organization (2022) defines mental health as a state of wellbeing that allows people to cope with stress, learn and work well, reach their capabilities, and contribute to their community. For children especially, a key component of mental health and wellbeing is social and emotional competence (Australian Institute of Health & Welfare, 2020). One in seven Australian children aged 4 to 17 years live with a mental health disorder and mental illness is the largest cause of disability and health burden in this age group (Australian Institute of Health & Welfare, 2020). This finding is also echoed in international literature, with a worldwide prevalence of mental health disorders in children of 13.4% (Polanczyk et al., 2015). The most common mental health diagnoses in this age group include attention deficit hyperactivity disorder (ADHD), anxiety, depression, and conduct disorders (Lawrence et al., 2015). While these data are the most recent prevalence statistics, international literature indicates higher rates of depression, anxiety, and post-traumatic stress symptoms experienced by children during the COVID-19 pandemic (Marques de Miranda et al., 2020) and US paediatricians and mental health experts predict long-term impacts on children’s mental health, especially for those already experiencing difficulties (Rider et al., 2021). Considering mental health difficulties in this age group are high and are likely increasing, it is important to understand the impacts this may have.

Children who experience poor mental health also show problems in social, behavioural, and emotional skills, and poor wellbeing. For example, children who may be struggling with their mental health (whether diagnosed or undiagnosed) can show a lack of social awareness and reciprocity, difficulties managing their emotions (e.g., outbursts of anger, expressing fear in non-threatening situations) and a range of behaviours that are either confrontational (violent and aggressive) or avoidant (e.g., not engaging in exploratory play) depending on the mental health problem (American Psychiatric Association, 2013). Such behaviours will potentially present and interfere with functioning in daily settings such as school and extended education, with educators required to identify and manage such behaviours as well as promote positive mental health amongst all children. It is therefore important for educators in extended education settings to have a good understanding of typical childhood development and mental health, to understand how mental health might impact daily behaviours, functioning and interpersonal relationships and to know how to support children.

### Wellbeing in Care Settings

Despite the large number of children enrolled in extended education internationally and the high prevalence of social, emotional, and behavioural difficulties in this age group, educators responsible for these children are not required to hold qualifications or formal training in child development, wellbeing, or mental health in Australia. Other countries qualifications requirements vary. Further, there are a lack of formal training programs developed specifically for Australian extended education settings (Cartmel & Brannelly, 2016; Cartmel et al., 2020), specifically those that train educators in how to recognise and support children with mental health difficulties. Thus, there is a need for research to identify solutions to improve educator capacity to manage children’s wellbeing and promote positive mental health in extended education.

The objective of this systematic literature review (SLR) is to determine what strategies and interventions have been tested in extended education settings to address or promote emotional, behavioural, or social wellbeing in children, and to assess how effective those strategies or interventions have been. Given this is an international issue and similar problems are evident worldwide, this SLR was conducted on international literature.

## Methods

A Preferred Reporting Items for Systematic reviews and Meta-Analyses (PRISMA; (Page et al., 2021) protocol was developed to guide the review and to maintain transparency. To address the objectives, several inclusion criteria were considered to ensure a relevant selection of studies were included. First, the studies must have been conducted in an extended education setting or with extended education staff to bring about change in extended education. Second, the studies must have introduced strategies or interventions designed to address or promote psychological constructs, including emotional, social, or behavioural wellbeing or development within a cohort of primary school-aged children (4–12 years). Studies were included if the tested strategies aimed to train or educate children in behavioural, social, or emotional wellbeing, or train educators in how to address or support the social, emotional, developmental, or behavioural wellbeing of children. Therefore, the population of interest was children and educators who work at extended education services. No restrictions were placed on gender, and studies from any country in the world were included if the article was accessible in English. Additionally, the studies must have examined the outcomes of the strategies, program, or intervention; specifically, the study must have included a measure of efficacy to show whether the program was effective. These outcomes could be educator-focused (e.g., educator levels of mental health and wellbeing literacy, self-efficacy and/or confidence), or child-/caregiver-focused (e.g., child or caregiver satisfaction with the intervention, changes in child wellbeing). This review included studies that measured qualitative or quantitative outcomes. Studies were excluded if they related only to primary school settings (without extended education context), those that focused on extended education itself as an intervention (i.e., the overall benefits to children attending extended education), and participants who were outside of the age range (i.e., under 4 years or over 12 years old).

### Search Strategy

The search string and strategy was determined in consultation with a Research Librarian. An electronic search was conducted in December 2023 with the following databases: EbscoHost Megafile, Scopus, SAGE Journals, Taylor & Francis, Web of Science, and Wiley Online Library. In September 2023 Australian and international extended education researchers were consulted at an extended education conference for additional relevant academic research to extended education and for clarification of search terms. Reference lists were hand searched to ensure inclusion of relevant studies.

The search terms were selected to include the four key constructs: after school care and names it is known by (“Outside Hours School” OR “After School Care” OR “After School Recreation” OR “School Age Care” OR “Out of School Hours” OR “School-Age Educare” OR “Extended Education”), mental health (“wellbeing” OR “well-being” OR “well being” OR “mental health” OR emotion* OR socio-emotional OR social OR behavioural OR develop*), the addition of an intervention (assess* OR strategies OR treatment OR program OR support OR education OR intervention), and the relevant age group of children (child* OR “primary school” OR “elementary school” OR “middle childhood”). After an initial search returned more than 50,000 articles, the search was narrowed to title and abstracts as they were determined to show the relevant results.

### Data Extraction

Two main reviewers firstly agreed on the clarity of terminology and the data to be collected before extraction. The reviewers assessed the relevancy of studies against the inclusion criteria independently at each phase of the literature search and agreed on results before moving to the next phase. A third reviewer was available to resolve any disagreements.

The following data were extracted from each of the included studies using an Excel data extraction template: title, author/s, publication year, country, study design, sample size, participant information, age range, participant gender, intervention name and description, delivery mode, location, target population, target skill/s, duration, frequency, who delivered the intervention and how, outcome measure, type of analysis, results of the study, limitations, and author notes (recommendations, comments, or concerns).

Due to the variation in study design and constructs targeted in each of the included studies, a meta-analysis was not deemed to be appropriate. Instead, a narrative synthesis was completed to provide a fusion of findings, explore relationships between the studies, assess robustness of the studies, and to group the findings by characteristics.

### Quality Evaluation

The mixed-methods appraisal tool (MMAT; Hong et al., 2018) was used to critically evaluate the quality of each study due to the variations in quantitative, qualitative, and mixed methods research designs of each. The MMAT was originally published in 2009 (Pluye et al., 2009) and has since been refined in 2011 (Pace et al., 2011) and in 2018 (Hong et al., 2018). Each version of the MMAT has undergone validity testing for interrater reliability, efficacy, and content validity, with the most recent showing excellent interrater agreement (*d* = 0.87–1.0) on the items (Hong et al., 2019).

The MMAT allows concurrent appraisal of the methodological quality of five research methods: qualitative, randomised control trial, non-randomised studies, quantitative, and mixed method studies. The first part is a screening checklist that asks, ‘are there clear research questions?’ and ‘do the collected data allow to address the research questions?’ The second part outlines five criteria assessing each of the five methodologies with responses of ‘Yes’, ‘No’, or ‘Can’t tell’.

As in the data extraction stage, two reviewers independently applied the MMAT criteria to each of the selected studies before discussing findings. A third reviewer was available to resolve any disagreements. The authors of the MMAT advise that sensitive analysis of the MMAT should take precedence over a simple tally; however, also recognise that reporting the results without a descriptive scoring system may be problematic (Hong et al., 2018). For this reason, an overall percentage score of each studies’ methodological quality will be offered along with a narrative analysis.

## Results

**Fig. 1:**
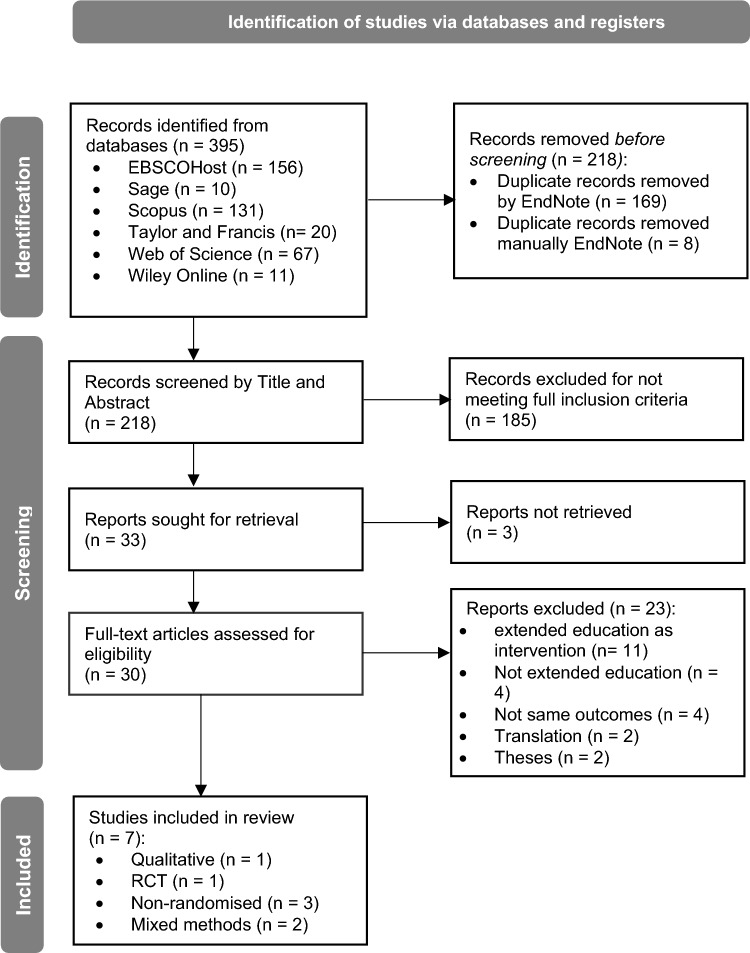
PRISMA flow diagram

Results of the search and screening process are presented in Fig. 1. The initial search returned 395 records. After duplicate records were removed, 218 records underwent title and abstract screening, with 185 records excluded at this stage for not meeting the full inclusion criteria. Articles were sought for full-text retrieval for further screening, and out of 33 full-text articles, 7 were selected for the final sample. Articles were most commonly excluded when the study investigated the extended education service as an intervention and there were no interventions introduced with the included outcomes. Due to naming conventions within Australia and internationally, four articles were excluded that did not conduct research in an extended education environment, typically because of the search string “out of school hours”. The PRISMA protocol flow chart is shown in Fig. 1 and details exclusion reasons at each step.

The seven included studies that comprise this review indicate the dearth of academic interest in children’s mental health and wellbeing outcomes in extended education settings and how educators understand and support these. Academic interest in this topic began less than 15 years ago (Bazyk & Bazyk, 2009), and half of the included studies occurred only in the past 5 years (Fettig et al., 2018; Minney et al., 2019; Siddiqui et al., 2019). A total of 1798 participants took part in the seven studies, although 1231 of these were from the one study (Siddiqui et al., 2019). Of the total participants, only 32 were adults (educators and volunteers; Milton et al., 2023) of the remaining, all were children and 967 of them participated in the interventions (the remainder were in control groups). This shows there were very few data gathered about educators, even in the three studies where the educators assisted in delivered the intervention to the children. Table 1 details the author, year, intervention cohort, intervention description, and intervention details and delivery for each of the included studies.

**Table 1:** Intervention details from selected studies

Author, year	Intervention cohort	Intervention description	Intervention details and delivery
Bazyk and Bazyk (2009)	African American low-income children attending a faith-based after school program in Ohio, USA	Healthy Occupations for Positive Emotions (HOPE) is an occupational therapy group designed to meet children’s need for structured leisure activities, social-emotional learning, and close human connection	Researchers implemented HOPE to all children at a service in 9 small (*n* = 7 or 8) sex segregated groups in 1 h sessions, once a week for 9 weeks. Sessions consist of 3 segments: • Conversation time (10–15 min) in which a social-emotional theme is introduced, • Activity (30–40 min) which include short-term (e.g., leather stamping) and long-term (e.g., paper-mache) projects, and • Closure (10 min) where children are encouraged to think about the relationship between doing the activities and how they feel
Fettig et al. (2018)	Students at risk for emotional and behavioural problems due to environmental factors, who attend an after-school program in the US	Super Friends, Super Readers (SFSR) is a language and social-emotional intervention that consists of dialogic story reading and social-emotional direct instruction	SFSR was offered by researchers to 4 children in 1 h weekly sessions over 6 months. Each session included: • Dialogic story reading (15–20 min) using scaffolded reading strategies, • Group activity (15 min) to extend and promote use of targeted social-emotional skill (e.g., role playing), and • Supervised free play (25–30 min) with limited adult support offered to children
Gooding (2010)	Children attending a US non-profit inner-city after school care program who were a mixture of typically developing and those with social, conduct, or behavioural deficits	Intervention uses CBT group therapy strategies incorporating music therapy curriculum delivered by a music therapist	Music therapy was delivered in a small group setting once a week for 5 weeks, in 45 min sessions. Each session included: • Introduction/social-emotional concept review (10 min), • New skill introduction and warm up activity (20 min), • Specific skill activity (10 min), and • Wrap up (10 min)
Kumschick et al. (2014)	Elementary aged children from 10 after-school centres in Germany were invited to participate	READING and FEELING aims to increase children’s emotional competence through reading and discussing a book with emotional content	Researchers read a book (Sheep with Boots by Matter (2003)) to small groups (*n* = 7) of children twice a week for 8 weeks, in 45 min sessions. Each session included: • Read a section of the book, • Discuss the identified emotional content, • Structured group activity (e.g., theatre), and • Individual quiet work (emotion diary)
Minney et al. (2019)	Elementary aged children attending an after-school care site at 6 locations in Texas, USA	A social-emotional learning (SEL) intervention was developed using a hybrid of other SEL curricula and adapted for an after-school program setting	Group leaders at the after-school program were trained and given materials each week to deliver to the children in 10–15 min sessions each day at the beginning of the after-school care program. This intervention ran for the whole year
Siddiqui et al. (2019)	Year 5 students at 68 schools across the UK participated	Children’s University (CU) is a charity trust that aimed to increase children’s aspiration, self-esteem, confidence, resilience, and social skills by providing outdoor activities and after school programs	Schools were funded to offer out of school activity groups in which children complete activities that are recorded in a passport. 15 h of activity must involve positive social action (community work, fundraising, etc.) and 15 h of non-specified extra-curricular activities
Milton et al. (2023)	Children at 5 OSHC settings in New South Wales, Australia	Connect, Promote, and Protect Program (CP3) is a programming framework to co-design activities aiming to: Connect Communities, Build Wellbeing and Resilience, Inspire and Engage, and Broaden Horizons	Stage 1: consult and create 1. Community consultation with families 2. Educator and volunteer workshops to train CP3 and build participatory design 3. Participatory design workshops with children 4. Workshopped activities selected Stage 2: Test and refine 3 activities (physical activity, creative pursuits, and skill development) chosen and tested over a few weeks during a school term Stage 3: Implement and evaluate over a school term with full cohort of children • E.g., Woodwork Café: trained CP3 mentors (volunteers and educators) worked with children (Connect Communities) and taught woodworking skills to build a chicken coop for the service (cognitive challenge and fun—Build Wellbeing and Resilience). A volunteer brought a chicken for a site visit, an OSHC family provided 2 baby chickens and incubator to hatch the eggs (Inspire and Engage) and children could engage in person or via the web to watch the chickens’ hatch (Broaden Horizons)

The methods and outcomes of each of the studies is examined in more detail in Table 2. With respect to the skills targeted in each intervention, most aimed to improve social-emotional skills (Bazyk & Bazyk, 2009; Fettig et al., 2018; Gooding, 2010; Minney et al., 2019), while the remaining three studies targeted a mixture of emotional, social, wellbeing, individual and community building skills (e.g., identifying masked feelings, teamwork, self-esteem, connect communities, etc.) (Kumschick et al., 2014; Siddiqui et al., 2019). The target population in each study was always children, however, some studies specified groups. For example, Bazyk and Bazyk’s (2009) study only included low socioeconomic African American children, Fettig et al. (2018) only included children at risk of emotional and behavioural problems, and Siddiqui et al. (2019) only offered the intervention to year 5 students.

**Table 2:** Method and effectiveness of selected studies

Author, year	Target skill	Participants	Study design/method	Outcome/effectiveness	Limitations
Bazyk and Bazyk (2009)	Aimed to identify and describe low-income children’s engagement in structured leisure activities that focused on group processes and social-emotional competencies	Purposeful sample of *N* = 10 from the group were interviewed • 60% female/40% male • 7–12 years old	Phenomenological qualitative study involving semi-structured time-series interviews (5 children interviewed 3 times, 3 interviewed twice, 2 interviewed once) between weeks 4–9 Also, random observation of 5 groups during sessions 4–8 for 10–30 min which focused on children’s occupational engagement, social interactions, group process, and setting • Interpretative phenomenological analysis	Researchers’ content analysis determined: 1. Groups are fun (occupational meaning) because of the new and creative activities in a supportive group (occupational form), which results in feeling happy and a desire to repeat the experience (occupational function), and 2. Children learned how to express feelings and respond to anger in healthy ways	• Didn’t directly measure whether SEL competencies improve • Small sample size • Limited language skills of the participants • No comparison group • Subject selection not randomised
Fettig et al. (2018)	Sought preliminary data on effectiveness of story reading on children’s language and SEL skills (turn-taking, problem solving, and positive peer feedback)	First 4 children whose completed paperwork was returned were selected from respondents to the study advertising • All males • 5–6 years old	Mixed-method case study • Quantitative: observers tallied the number of times SEL target skills were observed and successfully enacted in each session. Expressed as a percentage (e.g., number of child facilitated turn-taking divided by total opportunities to practice turn-taking, multiplied by 100) • Qualitative: content analysis of interviews with parents	Quantitative: • Interobserver agreement calculated to be 73% across all behavioural skills • Child facilitated problem solving success increased 7% • Turn taking increased 10.1% • Positive peer feedback increased 10.2% Qualitative: • Main theme of emotional regulation (increased ability to articulate problems and solve problems)	• Only 4 children were given the intervention • Researchers stated difficulty in live observation • Non-randomised subject selection
Gooding (2010)	Program targeted-specific social skill deficits: Peer relations (interpersonal skills, nonverbal communication, and verbal communication) and self-management skills	20 children were included in the study, *n* = 10 in control and *n* = 10 in intervention • 60% female/40% male • 6–11 years old (*M* = 8.5)	Randomised pre- post-mixed-method design • Children’s social competence was assessed by site program director rating on the social competence (SC) and antisocial (AS) subscales of the home and community social behavior scales (HCSBS), • Children self-rated using the Social Skills Assessment–Elementary Age (SSA), and • Researcher observations of the intervention group during sessions using the Group On-Task/Off-Task Teacher Response Form A (GOT/OT)	• HCSBS: Social competence scores improved in both groups over time - No sig. difference between groups in pre-test SC (*U* = 66, *p* > 0.05) and AS (*U* = 46.5, *p* > 0.05) or post-test SC (*U* = 42.5, *p* > 0.05) and AS (*U* = 50.5, *p* > 0.05) scores between groups • SSA: Sig. increase in both groups (*U* = 68, *p* (one-tailed) = 0.032) • GOT/OT: On task behaviour increased 10% from 1st session to last - Significant positive difference between 5 sessions, χ^2^ (12, 4) = 23.5, *p* = 0.000	• Small sample size • Researchers identified difficulty with inconsistent child attendance, space, and time constraints in extended education setting
Kumschick et al. (2014)	The intervention aimed to increase emotional vocabulary, the ability to identify masked feelings, explicit emotional knowledge, and the ability to discover mixed feelings	208 children were included in the study, *n* = 104 in control and *n* = 104 in intervention group • 62.5% female/37.5% male • 7–9 years old (T1 *M* = 7.94, SD = 0.72, T2 *M* = 8.13, SD = 0.72) • 6% attrition rate	Controlled quasi-experimental design with pre- post-test Educators allocated willing participants to control or intervention groups, with 15 groups of 7 children allocated to intervention. Both groups assessed on all measures pre- and post-intervention • Emotional competence (emotional vocabulary, explicit emotional knowledge, recognition of masked feelings, recognition of mixed feelings) assessed pre- and post-intervention by a board game designed for the study (laboratory of feelings) • Language competence (receptive vocabulary, literacy, verbal fluency, narrative abilities) assessed pre-intervention by individual 1.5 h language competence assessment with research assistants • Text analysis capability assessed by an emotion-in-text task after intervention to test expected increase of children’s ability to analyse emotional aspects of literature	Overall sig. positive correlation between age and language competence (*r* = 0.22, *p* < 0.01), and interaction of group and sex variables (group (*r* = 0.067, *p* < 0.01), sex (*r* = 0.53, *p* < 0.01) • Emotional competence: regression analyses showed children in test group sig. improved: - Emotional vocabulary (*B* = 1.25, *p* < 0.001) - explicit emotional knowledge (*B* = 1.32, *p* < 0.05), - Recognition of masked feelings (*B* = 1.05, *p* < 0.001) - No sig. improvement in recognition of mixed feelings • Text analysis capability: Between subject group and sex factors ANCOVA showed no difference between groups (*p* = 0.51), showing emotional literacy skills not evident when presented with a new book	• Unclear if the board game was a validated emotional measure • Group selection not randomised
Minney et al. (2019)	Three SEL skills were the focus of the study: self-management, self-awareness, and social awareness	125 children at 6 randomly selected after-school care sites completed both pre- and post-surveys and were included in the study • 50% female/50% male • 4–11 years old (*M* = 7.25, SD = 2.0)	Quasi-experimental single group pre- post-test design in which participants completed a researcher developed survey created for the study based on CASEL model – • Self-management (2 items) • Social awareness (2 items) • Self-awareness (1-item)	Statistically sig. increases in • Self-management (*p* < 0.001), and • Social awareness (*p* < 0.001) But not • Self-awareness (*p* = 0.339) • Medium effect size 0.50	• No control group included • Developed survey did not undergo validity testing • Self-report scales only provide partial understanding of skill improvement
Siddiqui et al. (2019)	Cognitive (math and reading) and non-cognitive (teamwork, communication, motivation, self-esteem, confidence, reliance, civic mindedness, and future intentions) skills were the focus of the study	68 schools were randomised to treatment (*n* = 36) or control (*n* = 32) and a total of 1231 children (654 in treatment, 557 in control) were included in this study Schools were able to offer the after-school program however they liked; 14 offered the program to the whole age cohort, 16 asked for volunteers, and 6 directed participation to the most disadvantaged students	• Baseline and post-intervention cognitive scores assessed by math and reading scores from the National Pupil Database • Author developed measure of non-cognitive skills using single items for each • Process evaluation to test intervention fidelity, implementation, and perceptions conducted by questioning while visiting schools • Intention to treat analysis with sub-analysis of disadvantaged students • Odds ratio to examine categorical variables	Headline findings: • Teamwork (ES = 0.02) and social responsibility (ES = 0.07) showed very small gains pre- to post-test • Larger gains seen by disadvantaged students in teamwork (ES = 0.17) and social responsibility (ES = 0.10) • Cognitive attainment effect sizes were small for treatment over control group between pre- and post-test for math (+ 0.15) and reading (+ 0.12) Increased participation of students from disadvantage was a key outcome	• Incomplete data reported in this publication • Mixture of random and non-random participant selection • Participation depended on school leadership • Issues highlighted by schools include lack of financial support, increased teacher workload, rural setting
Milton et al. (2023)	CP3 guiding principles are to 1) build wellbeing and resilience, 2) broaden horizons, 3) inspire and engage, and 4) connect communities	Children were the target population. Adults in the OSHC community were consulted *Formative:* • *N* = 16 children, 4–12 years old (*M* = 7.9, SD = 1.9), female 60.7%, male 39.3% • *N* = 32 adults (*n* = 10 educators, *n* = 6 volunteers, *n* = 9 parent/guardian, *n* = 7 other), 18–65 + years old, female 13.3%, male 86.7% *Process:* • *N* = 58 children, *M* = 7.9 years old (SD = 1.9) • *N* = 24 adults (*n* = 3 parent/guardians, *n* = 2 volunteers, *n* = 15 educators, *n* = 4 coordinators/managers)	Mixed method naturalistic formative and process evaluation of the CP3 program Formative quantitative survey: • Children only: language spoken at home, year at school • Adults only: relationship to OSHC site • All: gender, age, postcode, satisfaction with OSHC service, social connectedness (1-item Inclusion of Community in Self scale) and quality of life (Personal Wellbeing Index) Process quantitative: • Changes in educator completed Strengths and Difficulties Questionnaire at baseline and follow-up Both qualitative: • Six-step qualitative thematic analysis of surveys, interviews, and focus groups with adults and children	Formative: • Engagement was high: 10–15 children per session • Appropriateness and acceptability very high based on child, educator, and CP3 volunteer feedback. Children endorsed CP3 target areas between 84 and100% • Workplace satisfaction questions assessed feasibility, showed satisfied to very satisfied educators, with paperwork and being part of decision making at OSHC showing lowest satisfaction Process: • Effectiveness assessed by matched SDQ data (66%) showed 81% female, *M* = 7.9 (SD 1.9) years. Matched-sample *T*-test analysis found significant increase in prosocial behaviours (MD = 0.64, *p* = 0.04, t57 = − 2.06, 95% CI − 1.36 to − 0.02) and significant decrease in peer problems (MD = − 0.69, *p* = 0.01, t57 = 2.57, 95% CI 0.14–1.13) • No other significant changes in other scales Qualitative Themes included: • Program satisfaction • Child Outcomes: met all 4 CP3 principles • Educator and Volunteer Outcomes: provided professional development, increased job satisfaction, feelings of pride	• Evaluation only, target skills not the focus • Small sample size in formative evaluation • High attrition (52.5%) in matched samples

*CASEL* collaborative for academic, social, and emotional learning

Of the seven studies reported in this review, one employed qualitative methodology (Bazyk & Bazyk, 2009), three implemented non-randomised control trials (Kumschick et al., 2014; Minney et al., 2019; Siddiqui et al., 2019), one randomised control trial (Gooding, 2010), and two were mixed method studies (Fettig et al., 2018). Analyses varied in every study as shown in Table 2.

### Summary of Interventions

Only seven social and emotional or mental health interventions in extended education settings were found in the academic literature, highlighting the paucity of research. Of these, only three focused on creating extended education-specific interventions and delivered training to extended education educators themselves to support children’s social-emotional skills (Milton et al., 2023; Minney et al., 2019; Siddiqui et al., 2019). Reading stories with explicit SEL content was explored in two interventions which also focused on improving language and vocabulary through games, activities, and play (Fettig et al., 2018; Kumschick et al., 2014). While positive attainment of most of these skills was found, Fettig et al.’s (2018) participant population only included four children and Kumschick et al (2014) found no significant improvement in one SEL skill (recognition of masked feelings) and no between group differences when presented with a new book, indicating the measured improvements did not carry over to a new book.

Recognising a disparity in developmental outcomes for children in low socio-economic areas and a need for structured leisure activities outside of school time, Bazyk and Bazyk (2009), in the earliest example of introduced intervention, sought to improve low-income children’s social-emotional competencies through structured small group emotional and craft activities. While their content analysis of participant interviews showed themes indicating the groups were fun and children learned healthy ways to express feelings, there were no comparison groups or direct measurement of the stated skill improvements.

With a particular focus on disaffected youth and those who might be disadvantaged, Siddiqui et al (2019) evaluated the outcomes of the CU program, specifically focusing on cognitive and non-cognitive (i.e., SEL) skills building. Disadvantaged students showed greater attainment in non-cognitive skills of teamwork and social responsibility in this study. However, effect sizes for other non-cognitive and cognitive skills were small for all participants. Further, due to the variability of offerings for this intervention across multiple schools there was no further detail about which activities in particular showed greater skill attainment.

Gooding (2010) developed a CBT music therapy intervention delivered to small groups within extended education settings as well as schools and youth centres as part of a doctoral thesis. The aim was to improve children’s peer relation and self-management skills; however, while the children in this study showed improvements in these skills, so too did the control group, indicating the results may have been due to other factors such as child development more broadly.

The seven selected studies all trialled small group interventions designed to improve various social, emotional, and wellbeing skills of children already attending extended education settings. Four of the six interventions (Bazyk & Bazyk, 2009; Fettig et al., 2018; Gooding, 2010; Kumschick et al., 2014) in this review ran group sessions with the children that required a commitment of between 45 min and an hour and a half a week, for between 5 and 26 weeks. The children assigned to control in these studies participated in usual extended education activities. The intervention delivered by Siddiqui et al. (2019) is difficult to comment on due to the variations in the ways each school could offer the intervention. Minney et al (2019) and Milton et al. (2023) are the only interventions that trained the extended education educators themselves and offered all children in each of the extended education sites the opportunity to participate.

The researchers in the selected studies all highlighted extended education as an ideal environment to offer the designed interventions due to meeting inherent social, behavioural, and developmental needs. However, for most of these studies the extended education environment was not the focus of the intervention. Only Minney et al. (2019) and Milton et al. (2023) developed an extended education-specific intervention that was designed for adults to support all children who attend extended education.

### Quality Appraisal

The methodological quality of each included study was critically appraised using the MMAT, with the results displayed in Table 3. All but one study (Siddiqui et al., 2019) adhered to a high-quality methodological approach for their methodology type. While the authors of the MMAT do not recommend simply providing an overall score as it can mask problematic aspects of the study (Hong et al., 2018), a percentage rating is graphically depicted below. Four of the studies were rated at 80% or higher and were either randomised or non-randomised control trials. Interestingly, the only qualitative study was rated the highest in terms of methodological quality. The methodological quality of the studies averaged a rating of 71%.

**Table 3:** MMAT criteria for selected systematic literature review studies

Author, year	Qualitative					Randomised control trial					Non-randomized					Mixed methods					Quality score
	1.1	1.2	1.3	1.4	1.5	2.1	2.2	2.3	2.4	2.5	3.1	3.2	3.3	3.4	3.5	5.1	5.2	5.3	5.4	5.5	
Bazyk and Bazyk (2009)	Yes	Yes	Yes	Yes	Yes																100% (5/5)
Gooding (2010)						Yes	Yes	Yes	No	Yes											80% (4/5)
Kumschick et al. (2014)											No	Yes	Yes	Yes	Yes						80% (4/5)
Siddiqui et al. (2019)											Yes	Yes	No	No	No						40% (2/5)
Minney et al. (2019)											Yes	Yes	Yes	No	Yes						80% (4/5)
Fettig et al. (2018)																Yes	Yes	Yes	No	No	60% (3/5)
Milton et al. (2023)																Yes	Yes	No	Yes	No	60% (3/5)

1.1 = Are the qualitative data collection methods adequate to address the research question?; 1.2 = Is the qualitative approach appropriate to answer the research question?; 1.3 = Are the findings adequately derived from the data?; 1.4 = Is the interpretation of results sufficiently substantiated by data?; 1.5 = Is there coherence between qualitative data sources, collection, analysis and interpretation?; 2.1 = Is randomization appropriately performed?; 2.2 = Are the groups comparable at baseline?; 2.3 = Are there complete data?; 2.4 = Are outcome assessors blinded to the intervention provided?; 2.5 = Did the participants adhere to the assigned interventions?; 3.1 = Are the participants representative of the target population?; 3.2 = Are measurements appropriate regarding both the outcome and intervention (or exposure)?; 3.3 = Are there complete outcome data?; 3.4 = Are the confounders accounted for in the design and analysis?; 3.5 = During the study period, is the intervention administered (or exposure occurred) as intended?; 5.1 = Is there an adequate rationale for using a mixed methods design to address the research question?; 5.2 = Are the different components of the study effectively integrated to answer the research question?; 5.3 = Are the outputs of the integration of qualitative and quantitative components adequately addressed?; 5.4 = Are the divergences and inconsistencies between quantitative and qualitative results adequately addressed?; 5.5 = Do the different components of the study adhere to the quality criteria of each tradition of the methods?

## Discussion

This systematic literature review set out to examine the international literature to understand what, if any, interventions had been applied in extended education settings to improve children’s behavioural, social, and/or emotional wellbeing through either child or educator-focused training. A structured search and data collection following the PRISMA protocol found only seven international studies met the inclusion criteria. These seven studies provided a mixture of methodologies which consisted of varying designs and outcomes. This review highlights the lack of clarity in regard to which interventions are known to promote social, emotional, behavioural skills or mental health in extended education contexts. There was evidence to support programs designed specifically for extended education when services and educators are involved in intervention delivery. While the interventions varied significantly between studies, the cohort target, outcomes, and purpose of each intervention showed considerable homogeneity across studies in this review.

### Strength of Evidence for Interventions

While the quality of these studies was generally rated highly as appraised by the MMAT (Hong et al., 2018), the overall strength of evidence was relatively weak, due to the lack of studies overall, and the lack of Clinical trials conducted. For the interventions that showed positive effects, only Level 2.c evidence (quasi-experimental prospectively controlled study designs) was provided for the CU program (Siddiqui et al., 2019), reading groups with feelings board game (Kumschick et al., 2014), and for the whole service small groups intervention delivered by educators (Minney et al., 2019). Level 3.e evidence (observational study without a control group design) was provided for occupational therapy groups (Bazyk & Bazyk, 2009), CP3 program (Milton et al., 2023), and the dialogic story reading intervention (Fettig et al., 2018). This systematic review has therefore indicated a gap in the literature for high-quality, evidence driven interventions and Clinical trials.

Based on the evidence to date, no interventions have been identified to specifically develop children’s behavioural wellbeing or mental health in extended education contexts. All interventions focused on SEL competencies and results of this review show that the educator delivered small group intervention which targeted self-management and social awareness skills was most effective (Minney et al., 2019). This study also demonstrated the highest level of evidence for this review (The Joanna Briggs Institute, 2014) and a high-quality MMAT rating (Hong et al., 2018). Table 4 provides comparisons of the methodological quality and level of evidence for each intervention, as well as how well the outcomes were achieved and ranks them in order of efficacy.

**Table 4:** Comparison of included interventions in order of efficacy

Intervention	MMAT (%)	JBI Levels of Evidence	Positive changes	Did not change
Extended education SEL groups (Minney et al., 2019)	80	Level 2.c	Self-management and social awareness	Self-awareness
Dialogic reading interventions (Kumschick et al., 2014)	80	Level 2.c	Emotional vocabulary, explicit emotional knowledge, recognition of masked feelings	Recognition of mixed feelings
CP3 (Milton et al., 2023)	60	Level 3.e	Prosocial behaviours and decreased peer problems	Emotional symptoms, conduct problems, hyperactivity
SFSR (Fettig et al., 2018)	60	Level 3.e	Turn taking, problem solving, positive peer feedback	
CU (Siddiqui et al., 2019)	40	Level 2.c	Teamwork, social responsibility	
OT leisure activities (Bazyk & Bazyk, 2009)	100	Level 3.e		Did not measure SEL
CBT music therapy (Gooding, 2010)	80	NA		No sig. differences between groups

The only other intervention with positive outcomes, but lower strength of overall evidence was the dialogic reading intervention (Kumschick et al., 2014). CP3 (Milton et al., 2023), SFSR (Fettig et al., 2018), and CU (Siddiqui et al., 2019) provided limited overall evidence; however, it should be noted that CP3 was only in the evaluation phase of research and the outcomes have not yet been explicitly tested. The remaining two interventions were considered as providing limited evidence despite high-quality methodological designs as they either did not show positive effects in social competence or social skills (Gooding, 2010) or did not provide evidence of increased skills (Bazyk & Bazyk, 2009).

### Educators

There appears to be an almost complete absence of research examining interventions delivered to or by educators with the results of this review showing all interventions delivered in extended education settings were designed to be delivered only to children. In all but three interventions (Minney et al., 2019; Siddiqui et al., 2019), educators did not deliver the interventions and did not receive support either during or after the research. For the studies that did include educators delivering the interventions, only Milton et al. (2023) collected some baseline details; however, none sought information regarding their ability or effectiveness in supporting children’s social, behavioural, or emotional wellbeing. In the limited research that exists, only two interventions Milton et al. (2023) and Minney et al. (2019) attempted to upskill educators in their ability to manage children’s social-emotional, behavioural, or mental health knowledge. Thus, future research would benefit from focusing on this specific area.

The recent MTOP update explicitly discusses the mental health of children and increases the provision for educators to provide for the wellbeing of children in their care (Department of Education for the Ministerial Council, 2022). ‘Outcome 3: Children and young people have a strong sense of wellbeing’ now highlights educators’ unique position to support children’s mental health and wellbeing through attuned care and by creating child safe cultures and environments appropriate to their development and needs (Department of Education for the Ministerial Council, 2022). It will be important that extended education services and educators can demonstrate adherence to these updates.

### Extended Education as an Intervention

Although this review found only seven studies examining interventions implemented in extended education settings, there has been international interest in the social and emotional outcomes associated with extended education attendance in general. The consensus of this mostly US-based quantitative literature shows many positive SEL outcomes from attending extended education (e.g., Durlak et al., 2010); however, extended education takes many forms internationally. For example, a meta-analysis of services that offer activities after school in the US found that the 52 included extended education programs showed a significant positive effect on children’s feelings, behaviour, and attitudes to school (Durlak et al., 2010) As this analysis investigated studies of all after school activities, many of the included interventions were not conducted in a typical extended education setting (e.g., drug prevention program, Little League, etc.) or evaluated extended education as the intervention and therefore did not meet the inclusion criteria of our study, but provides positive support for wellbeing promotion in these settings. Highlighting the difficulties conducting research in this area, Durlak et al. (2010) discussed the lack of equivalence between services, making it difficult to make meaningful comparisons. Further, considering only one of the seven studies used qualitative methodology (Bazyk & Bazyk, 2009), this should be a focus in future research.

### Limitations

This study was limited by the lack of peer reviewed literature examining introduced social-emotional and mental health support in extended education settings. Until recently, this has not been considered in the literature and highlights a gap in the research. Research in Australia has been particularly lacking; however, it is positive to see work is beginning in the area. Second, the heterogeneity of included interventions prevented meaningful overall data comparisons or conclusions. This again highlights the importance of research to provide aggregated data that will support effective mental health interventions in extended education. Third, only peer reviewed research was included in the search criteria and does not account for any informal interventions introduced in extended education or any unpublished research in progress. Finally, although extended education researchers in Australia were consulted for alternative terms for extended education or conceptions of mental health and wellbeing, there may be search terms that have been unintentionally overlooked.

## Recommendations and Conclusion

This is the first systematic review to investigate interventions to improve social, emotional, and/or behavioural wellbeing in extended education settings. Children were the target cohort of all the seven interventions, with the studies highlighting various ways to increase social and emotional skills, but not behaviour skills or mental health directly. While training educators in the delivery of social and emotional content showed promise in teaching children self-management and social awareness skills, none of the studies examined educators’ capability and self-efficacy to deliver any such interventions in extended education. Given the dearth of studies identified, future research should focus on conducting high-quality trials of various interventions to improve SEL and mental health in extended education contexts. Future research should also consider educators’ knowledge of, capability, and confidence to support children’s mental health and wellbeing in these settings. Research should include qualitative data (such as interviews or open ended questions) to ensure educator and mental health expert input to developing strategic supports and interventions. There is an urgent need for more research into strategies and interventions to promote positive emotional and behavioural development and mental health within school-aged children via extended education contexts.

## Data Availability

The data that supports the findings of this study are available on the Harvard Dataverse website: 10.7910/DVN/6W79DK.
